# Advances in research and application of artificial intelligence and radiomic predictive models based on intracranial aneurysm images

**DOI:** 10.3389/fneur.2024.1391382

**Published:** 2024-04-17

**Authors:** Zhongjian Wen, Yiren Wang, Yuxin Zhong, Yiheng Hu, Cheng Yang, Yan Peng, Xiang Zhan, Ping Zhou, Zhen Zeng

**Affiliations:** ^1^School of Nursing, Southwest Medical University, Luzhou, China; ^2^Wound Healing Basic Research and Clinical Application Key Laboratory of Luzhou, School of Nursing, Southwest Medical University, Luzhou, China; ^3^School of Nursing, Guizhou Medical University, Guiyang, China; ^4^Department of Medical Imaging, Southwest Medical University, Luzhou, China; ^5^School of Basic Medical Sciences, Southwest Medical University, Luzhou, China; ^6^Department of Interventional Medicine, The Affiliated Hospital of Southwest Medical University, Luzhou, China; ^7^Department of Radiology, The Affiliated Hospital of Southwest Medical University, Luzhou, China; ^8^Psychiatry Department, The Affiliated Hospital of Southwest Medical University, Luzhou, China

**Keywords:** radiomics, artificial intelligence, machine learning, deep learning, intracranial aneurysm

## Abstract

Intracranial aneurysm is a high-risk disease, with imaging playing a crucial role in their diagnosis and treatment. The rapid advancement of artificial intelligence in imaging technology holds promise for the development of AI-based radiomics predictive models. These models could potentially enable the automatic detection and diagnosis of intracranial aneurysms, assess their status, and predict outcomes, thereby assisting in the creation of personalized treatment plans. In addition, these techniques could improve diagnostic efficiency for physicians and patient prognoses. This article aims to review the progress of artificial intelligence radiomics in the study of intracranial aneurysms, addressing the challenges faced and future prospects, in hopes of introducing new ideas for the precise diagnosis and treatment of intracranial aneurysms.

## Graphical Abstract

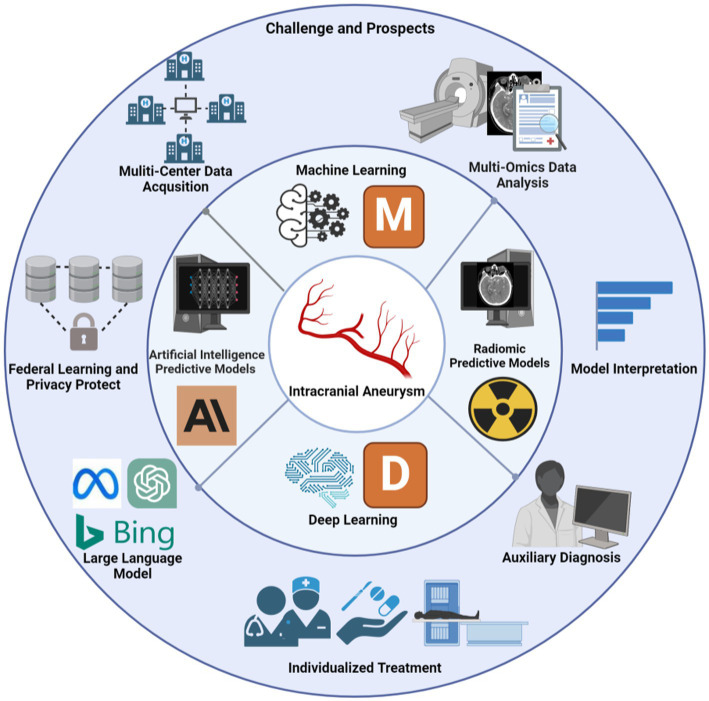

## 1 Introduction

Intracranial aneurysms (IAs) are a type of cerebrovascular disorder primarily caused by congenital defects in the cerebral artery walls or internal pressure, leading to abnormal dilation and protrusion of the artery walls (1). Globally, the prevalence of IAs is estimated to be between 3 to 5%, while in China, it is about 7%, significantly impacting patient outcomes (2).

Imaging examinations play a pivotal role in the early screening of IAs. Digital subtraction angiography (DSA) is considered the gold standard for IA diagnosis; however, its invasive nature, high risk, and cost limit its clinical application (3, 4). With the continuous development of imaging technology, non-invasive methods like CT angiography (CTA) and magnetic resonance angiography (MRA) are increasingly used for IA detection (5). CTA, known for its non-invasiveness and convenience, has become a primary method for vascular lesion screening, though it does not provide hemodynamic information of aneurysms (6). MRA, characterized by its non-ionizing nature and excellent tissue resolution, can offer information on blood flow conditions (7). Techniques like 4D flow MRI based on magnetic resonance can quantitatively obtain multi-directional hemodynamic parameters within arteries, and high-resolution MRI vessel wall imaging (HRMR-VWI) with its high tissue resolution can clearly depict the structure of vessel and aneurysm walls, assessing inflammatory and pathological changes based on aneurysm wall enhancement features (8).

Currently, the detection of IAs relies heavily on the experience of physicians, with CTA and MRA facing challenges in diagnosing small aneurysms, posing a risk of missing them. In the clinical treatment and decision-making process for IAs, clinicians often determine treatment plans by combining patients’ clinical characteristics and individual circumstances, making it difficult to directly use imaging information for decision-making support. With the rapid advancements in artificial intelligence and imaging technologies in recent years, AI and radiomics have been widely applied in early disease screening, status assessment, and prognosis prediction using medical imaging data, offering new possibilities for clinical detection and personalized precision treatment of IA patients. This study summarizes the application of deep learning and radiomics in the clinical diagnosis, status assessment, and prognosis prediction of IAs, proposing new approaches to address current challenges.

## 2 Overview of artificial intelligence and radiomics

### 2.1 Workflow of a radiomics study

**Figure 1:**
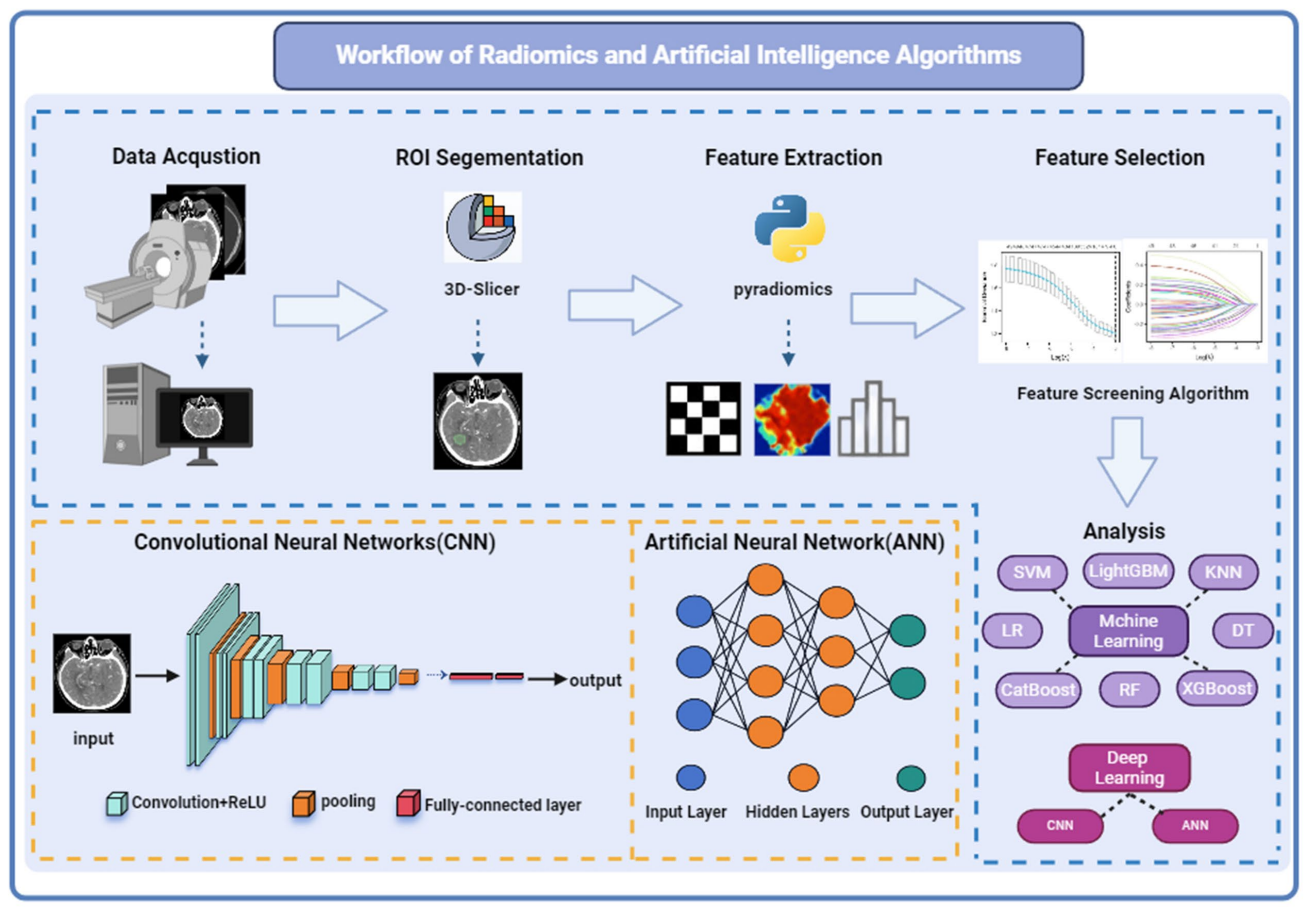
Workflow and principles of radiomics and artificial intelligence algorithms. Referenced and reproduced with permission from (1).

The general workflow of radiomics and artificial intelligence is shown in Figure 1. Radiomics aims to extract and analyze patient imaging data, uncovering medical imaging features such as shape, size, texture, and intensity, including perianeurysmal enhancement, that are difficult to discern with the naked eye but can reveal the microstructure of lesions (9). Radiomics data can be integrated with patients’ clinical data to identify biomarkers and correlations in imaging that indicate patient prognosis and lesion status. The workflow of radiomics generally includes: (1) image acquisition (2) dataset creation: typically comprises clinical data and imaging material (3) image preprocessing: generally includes image denoising, normalization, and enhancement (4) delineation of the region of interest (ROI): often done through expert manual annotation (5) extraction of radiomic features: includes texture features, intensity features, etc. (6) feature selection and dimensionality reduction: commonly employed techniques include regression models for supervised learning or clustering models for unsupervised learning (7) model development and validation: typically utilizes machine learning and deep learning methods applied to binary or multi-class tasks, with model predictive results applied to external validation datasets to ensure the model’s generalizability.

### 2.2 Overview of artificial intelligence

Machine learning (ML), a key research method in radiomics within artificial intelligence technology, predicts the occurrence of an outcome event by analyzing radiomics feature, capable of autonomously learning to recognize complex patterns within large and intricate datasets. Machine learning can be divided into supervised learning, unsupervised learning, and reinforcement learning. Supervised learning includes random forests, support vector machines, KNN, XGBoost, LightGBM, etc. Supervised learning requires manually labeled training data and trains the optimal model through known relationships, commonly used for classification and regression. Common unsupervised learning methods include k-means clustering. Unsupervised learning does not require labeled data and can directly analyze data through assumptions. It is mainly used for clustering and dimensionality reduction. Reinforcement learning focuses on how to take actions in an environment to maximize some cumulative reward. This learning process is based on the interaction between the agent and the environment, with the core being learning a policy, that is, mapping from states to actions, to optimize long-term rewards. In addition, machine learning also includes other complex learning types, such as semi-supervised learning and weakly supervised learning, which train on incompletely labeled datasets to overcome the problem of high data labeling costs in traditional supervised learning.

In deep learning algorithms, CNNs, ANNs, and backpropagation neural networks are all applied in the study of intracranial aneurysms, among which CNNs are the most widely used. CNNs identify and extract key features in images through convolutional and activation layers. Pooling layers further reduce the feature dimensions, decreasing the computational burden of the model. The fully connected layers integrate these features to provide a basis for the final decision-making. During the entire training process, backpropagation and optimization algorithms (such as gradient descent) are used to continuously adjust network parameters to minimize prediction errors (10). CNNs learn discriminative features from input data through convolution, pooling, and activation steps, constructing feature hierarchies from low to high levels (2, 11). At lower levels, the network learns relatively simple features like lines and edges (12). At higher levels, CNNs can combine these simple features into complex patterns, such as specific shapes or objects (13). CNNs can also enrich data composition by extracting deep learning features through convolutional layers and combining them with radiomic features (14). Radiomics based on convolutional neural networks (CNNs) differs from traditional radiomics in that the models can automatically learn to extract and select image features. Therefore, it does not introduce additional errors in radiomic analysis due to feature computation, ensuring the accuracy and effectiveness of image features (15).

Large language models (LLMs) represent another major branch in the AI field and have recently garnered widespread attention in the field of radiology. The Transformer, foundational to the development in the LLM domain, is essentially a deep neural network based on a self-attention mechanism, mainly comprising encoders and decoders. The Transformer model efficiently captures dependencies between different positions within sequence data and processes sequences of natural language data of certain lengths (16). Due to its independence from extensive manual annotations and the ability to handle multimodal data, Transformers are considered to have more potential than traditional CNNs. However, Transformers generally have a larger number of parameters and require extensive data for training. In contrast, CNNs capture local features through convolutional layers and implement parameter sharing, maintaining good performance even with small sample sizes. Therefore, in some scenarios, combining the strengths of CNNs and Transformers could be an effective strategy.

## 3 Applications

### 3.1 Detection and diagnosis of intracranial aneurysms

Most patients with unruptured intracranial aneurysms (IAs) do not exhibit noticeable symptoms. The hidden nature and morphological diversity of IAs often lead to missed diagnoses of small aneurysms. Trained convolutional neural networks (CNNs), with their multi-layered structures, can learn and recognize the complex morphological features of IAs across all imaging modalities, performing precise detection and segmentation to separate aneurysm areas from surrounding brain tissue and vessels. Therefore, the automatic segmentation and diagnosis of IAs have become research focal points, widely applied across imaging modalities such as CTA, MRA, and DSA. However, AI’s challenges in identifying small aneurysms (less than 3 mm) and misdiagnosing the remnants of vessel occlusions as aneurysms highlight the technology’s significant limitations in clinical practice. Currently, these technologies are mostly in the development stage and have not been widely implemented in clinical settings (see Table 1).

**Table 1:** A brief summary of papers on the application of artificial intelligence and radiomics in the automated detection and segmentation of intracranial aneurysms.

Author and year	Imaging modality	Dataset size and source	Methodology	Model validation methods	Clinical outcomes predicted	Evaluation metrics
Ueda et al. (17)	MRI	683 patients, multicenter	ResNet-18	Internal and external validation	Detection and segmentation	Internal validation: TPR = 91% External validation: TPR = 93%
Claux.et al. (18)	3D TOF-MRA	49 patients, single center	U-Net	Internal validation	Detection and segmentation	TPR = 78%
You et al. (19)	CTA	3,190 patients, multicenter	U-Net	Internal and external validation	Detection and segmentation	Internal validation: DSC = 0.78, 0.71, 0.71 External validation: DSC = 0.66 Internal validation: TPR = 98.58,95.00, 96.00% External validation: 96.17%
Zhu et al. (14)	CTA	101 patients, single center	3D U-Net, V-Net, 3D Res-Unet	Internal validation	Detection and Segmentation	3D U-Net: DSC = 0.818 ± 0.100
Hu et al. (26)	CTA	17,277 patients, multicenter	GCN + LFN	Internal and external validation	Detection	Internal validation: TPR = 95.7% External validation: TPR = 98.8%
Chen et al. (20)	3D TOF-MRA	1,160 patients, multicenter	Dual-channel SE-3D U-Net	Internal and external validation	Detection	Internal validation: TPR = 82.46%
Jin et al. (2)	2D-DSA	347 patients, single center	End-to-end spatiotemporal deep neural network	Internal validation	Detection and segmentation	DSC = 0.533, TPR = 97.7%
Duan et al. (11)	2D-DSA	281 patients, single center	Cascade CNN based on FPN with ResNet50 as the backbone	Internal validation	Detection	AUC = 0.942
Podgorsak et al. (21)	2D-DSA	350 patients, single center	CNN and radiomics	Internal validation	Detection and segmentation	DSC = 0.903 AUC = 0.791
Bizjak et al. (6)	MRA和CTA	3,228 patients, multicenter	U-Net and Point Net++	Internal and external validation	Detection	External validation MRA: TPR = 85% CTA: TPR = 90%
Sichtermann et al. (25)	3D TOF-MRA	85 patients, single center	Deep-medic based on CNN	Internal validation	Detection	TPR = 90%

DSC, Dice similarity coefficient; TPR, sensitivity; AUC, area under curve; GCN, global context network; LFN, local fine-grained network; CNN, convolutional neural networks.

#### 3.1.1 Intracranial aneurysm identification and diagnosis based on digital subtraction angiography images

To explore the detection performance of CNNs for IAs, Jin et al. (2) trained a network on 2,269 DSA sequences from 347 IA patients using a generic U-Net convolutional network design tailored for medical image segmentation and detection. The network incorporated bidirectional convolutional long short-term memory modules at each level to capture changes in contrast agent flow within 2D DSA images, allowing the convolutional network to contain both spatial and temporal information from the DSA sequences for end-to-end training. The results demonstrated a patient-level sensitivity of 97.7%. Duan et al. (11) introduced a cascaded CNN architecture based on feature pyramid networks (FPN) with ResNet50 as the network backbone. In the first stage, which is the global localization stage, the posterior communicating artery is located from 2D-DSA images. In the second stage, dual inputs merge two images of the posterior communicating artery output from the global localization stage and input them into the second-stage FPN to detect intracranial aneurysms. The study indicates that the dual-input model is more stable under different data conditions than the single-input model. Compared with automatic computer-aided diagnosis based on classical digital image processing (DIP) methods for DSA modality, the results showed that the proposed architecture has an accuracy of 93.5% and an AUC of 0.942, which are significantly higher than the classical DIP methods. Moreover, the CNN detection time required is only 1% of that required by DIP methods. Those studies highlight the superior detection capabilities of various CNN architectures on DSA images for IAs, but CNNs cannot utilize the relational and informational context of each frame in an entire DSA sequence, which may affect detection accuracy. Existing research has shown that recurrent neural networks (RNNs) can integrate contextual information from time-series data (12). Future research could explore combining RNNs with CNNs, utilizing RNNs to convey features extracted by CNNs from DSA sequences, leveraging the temporal context of DSA sequences to achieve higher precision in predictions for medical imaging data with complex temporal dynamics.

#### 3.1.2 Detection and segmentation of images based on CT angiography and magnetic resonance techniques

To verify the performance of deep learning in the detection and segmentation of intracranial aneurysm (IA) images from CT angiography (CTA) and MR, Ueda et al. (17) proposed an 18-layer CNN residual network architecture for MRI images, achieving sensitivities of 91% (internal dataset) and 93% (external dataset). Claux et al. (18) employed a dual convolutional neural network based on a regularized U-Net architecture to enhance performance in situations with limited training data. The results demonstrated a sensitivity of 78% and a positive predictive value of 62%. The dual convolutional model was able to achieve accurate intracranial artery segmentation and effective aneurysm detection on 3D TOF MRA images. These studies showcase the promising performance of artificial intelligence technologies in MR imaging.

You et al. (19) developed a novel U-net network that incorporates a vascular attention model, trained on CTA images from 3,190 patients with intracranial aneurysms. The results demonstrated that the internal test set sensitivity reached 95–96%, and a sensitivity of 96.17% was achieved in the external validation set. The model also achieved more robust segmentation performance, with Dice scores of 0.71–0.78 on the internal test set and 0.66 on the external test set. The obtained model has higher segmentation accuracy than previously developed deep learning algorithms, showcasing the superior performance of artificial intelligence in CTA image detection and segmentation. Although this study included the largest and most complex dataset to date, the lack of negative samples without aneurysms in the dataset may affect the false positive rate due to sample imbalance. Therefore, establishing a comprehensive and standardized dataset remains an important goal.

Zhu et al. (14) used small sample datasets to compare the detection of aneurysms using 3D UNet, VNet, and 3D Res-UNet, with 3D UNet showing the best detection performance. This suggests that the architectural structure of different CNN models could impact detection and segmentation capabilities. Chen et al. (20) developed an aneurysm detection model based on a dual-channel SE-3D UNet, retrospectively collecting 1,096 TOF-MRA images of unruptured intracranial aneurysms. The model, which divided the dataset into training and validation sets chronologically, outperformed the basic SE-3D UNet, increasing patient-level sensitivity by 15.79% and reducing false positives by 4.1%. These findings emphasize the importance of continuous exploration and integration of the latest AI technologies.

Beyond direct CNN application for IA imaging detection and segmentation across various modalities, Podgorsak et al. (21) combined deep learning with radiomics, automating the extraction of radiomic features through CNNs for IA detection and segmentation. While deep learning (DL) enables fully automated analysis of imaging post-model training, it requires significantly larger data volumes compared to radiomics, and the data volume in most studies is limited. Therefore, combining DL radiomics (DLR) features with clinical parameters or classical radiomics features can reduce the demand for large sample sizes, enhancing the accuracy and reliability of classification or prediction outcomes. This approach offers potential assurance for optimizing personalized diagnosis and treatment.

Despite promising results in automated detection studies, current capabilities still show significant room for improvement, especially in detecting small and very small aneurysms (22, 23). This is related to the subtle imaging features of tiny aneurysms, which are easily overlooked or confused with normal physiological structures, preventing the effective capture of their characteristics. Research has found that compared to regular aneurysms, small aneurysms with a diameter of <5 mm have a higher proportion of severe cases classified under Fisher’s scale for subarachnoid hemorrhage caused by aneurysm rupture, and the proportion of poor prognosis is not significantly different from other aneurysms (24). Therefore, to ensure patient safety and quality of life, it is essential to develop new detection technologies to improve the detection capability for tiny intracranial aneurysms (IAs). Bizjak et al. (6) introduced a novel IA detection method based on deep geometric learning, applicable to both MRA and CTA images and validated across multiple internal and external datasets, showing sensitivities of 72 and 83% for detecting small aneurysms in MRA and CTA images, respectively.

Artificial intelligence models have an absolute advantage in processing speed compared to doctors and can provide effective assistance to radiologists with less clinical experience. Sichtermann et al. (25) compared the sensitivity of two professional doctors in identifying aneurysms independently and with the assistance of the DeepMedic CNN model, confirming improved detection sensitivity under CNN-assisted diagnosis. Hu et al. (26) established the largest dataset of intracranial aneurysm CTA images to date, incorporating CTA images of 14,517 patients for AI model development and internal validation, and retrospectively collected data from 1,198 patients for external validation, conducting prospective studies in five hospitals. The study showed that the AI model achieved high diagnostic accuracy in the external validation set (sensitivity of 98.8%, specificity of 81.2%, and negative predictive value of 99.8%), surpassing the performance of radiologists, with an error rate of 0.5% in prospective validation. AI-assisted reading significantly improved the diagnostic performance of clinical doctors, with the AUC increasing from 0.787 to 0.909 and patient-level sensitivity from 0.590 to 0.825, particularly enhancing the diagnosis of aneurysms <3 mm by 31.8%, reducing the probability of missed diagnoses. This aids in early screening and the development of personalized treatment plans for patients, improving patient prognosis. The study highlighted that for less experienced resident doctors, utilizing deep learning models for assisted diagnosis can yield more significant benefits. However, the study was limited to CTA images, and there is currently a lack of comparative discussions and prospective validation of AI applications in DSA, MRI, and other areas. Further repeated and large-sample studies are needed to ascertain the practical value of AI models in clinical application.

### 3.2 Recognition and prediction of IA status

Subarachnoid hemorrhage (SAH) caused by the rupture of intracranial aneurysms (IAs) often results in extremely high mortality and morbidity rates, with survivors frequently suffering from long-term neurological sequelae that diminish their quality of life (27). The clinical manifestations of IA rupture are complex, primarily due to the aneurysm’s structural morphology and location. Some patients with ruptured IAs do not experience SAH, resulting in relatively good prognoses. On the other hand, locating the rupture site in patients with multiple aneurysms, even in the presence of SAH, can be challenging, making accurate recognition of IA status crucial for clinical treatment and prognosis assessment (28, 29). In clinical practice, healthcare professionals still widely use scale scoring methods to predict the risk of aneurysm rupture, such as PHASES score, UIATS score, and ELAPSS score. However, studies comparing machine learning models (including SVM, RF, and ANN) with traditional statistical models and the PHASES score have confirmed the superior performance and application potential of machine learning algorithms in predicting intracranial aneurysm rupture (30). Yang et al. (31) conducted a prospective study on IA rupture risk assessment using a backpropagation (BP) neural network and compared the predictive performance of BP, PHASES, UIATS, and ELAPSS scores through ROC analysis. The results showed that BP outperformed PHASES and ELAPSS scores but was slightly inferior to UIATS, which may be related to the smaller sample size included in the study. The research also indicated that using UIATS carries the risk of overtreatment, whereas artificial intelligence algorithms can effectively reduce the probability of overtreatment. This not only benefits the physiological health of patients but also helps avoid unnecessary economic burdens and psychological stress. The superiority of artificial intelligence in predicting aneurysm rupture may be due to the complex and nonlinear relationships between clinical manifestations and data. For instance, the rupture of intracranial aneurysms is often the result of interactions among multiple factors. Therefore, compared to traditional statistical models (such as logistic regression) and scale scoring, which are limited to linear relationships, artificial intelligence algorithms can better capture these nonlinear relationships by constructing multi-level data representations from simple to complex, quickly providing more accurate results (see Table 2).

**Table 2:** A brief summary of papers on the application of artificial intelligence and radiomics in the detection and prediction of intracranial aneurysm status.

Author and year	Imaging modality	Dataset size and source	Methodology	Model validation methods	Clinical outcomes predicted	Evaluation metrics
Zhu et al. (30)	3D-DSA	2,179 patients, single center	ML (SVM, RF, ANN)	Internal validation	Stability assessment	SVM: AUC = 0.858 RF: AUC = 0.850 ANN: AUC = 0.867
Liu et al. (32)	3D-DSA	368 patients, single center	Radiomics and ML (glm-lasso)	Internal validation	Stability assessment	AUC = 0.854
Yang et al. (34)	CTA	576 patients, multicenter	Radiomics and ML (AdaBoost, XGBoost, CatBoost)	Internal validation	Identification of status	AdaBoost: AUC = 0.889 XGBoost: AUC = 0.883 CatBoost: AUC = 0.864
Feng et al. (35)	CTA	363 patients, multicenter	Radiomics, CNN and ML (SVM, RF, MLP)	Internal and external validation	Identification of status	Internal validation: SVM = 0.86 RF = 0.85 MLP = 0.90 External validation: SVM = 0.85 RF = 0.88 MLP = 0.86
Xie et al. (36)	CTA	106 patients, single center	Radiomics, CNN and ML(SVM)	Internal validation	Stability assessment	AUC = 0.8909
Turhon et al. (37)	3D-DSA	1,740 patients, multicenter	Radiomics, DL(Transformer) and ML(SVM)	Internal and external validation	Stability assessment	Internal validation: DL: AUC = 0.929 External validation: DL: AUC = 0.823
Chen et al. (39)	3D-DSA	148 patients, single center	DL(PointNet) and ML (RF, KNN, XGBoost, LightGBM)	Internal validation	Stability assessment	AUC_MAX_ = 0.969
Li et al. (40)	CTA	423 patients, single center	Trans IAR-net	Internal and external validation	Stability assessment	Internal validation: AUC = 0.9224 External validation: AUC = 0.9803

AUC, area under curve; CNN, convolutional neural networks; MLP, multi-layer perceptron; ANN, artificial neural network; RF, Random Forest; SVM, Support Vector Machine; AdaBoost, Adaptive Boosting; XGBoost, eXtreme Gradient Boosting; CatBoost, Categorical Boosting; KNN, K-Nearest Neighbors; LightGBM, Light Gradient Boosting Machine.

#### 3.2.1 Prediction of IA rupture based on radiomics

The use of morphological characteristics of intracranial aneurysms to predict aneurysm rupture has received extensive validation. Liu et al. (32) segmented aneurysms using 3D Slicer, selecting 420 aneurysms with maximum 3D diameters between 4 mm and 8 mm for analysis. Innovatively, they employed the Python-based “PyRadiomics” package to extract 12 derived morphological features, including shape, size, and surface area, and integrated these with clinical features to construct two machine learning models: glm_step and glm_lasso. The glm_lasso model was identified as superior, with an AUC of 0.853. However, a study by Ludwig et al. (33) found that radiomics-derived morphological features extracted from DSA did not significantly enhance the predictive performance for aneurysm rupture, contradicting Liu’s assertion that flatness is the most critical morphological determinant for predicting aneurysm stability. Yang et al. (34) utilized radiomics features to distinguish between ruptured and unruptured intracranial aneurysms in the middle cerebral artery, constructing classification models with 12 common machine learning algorithms. The models built on AdaBoost, XGBoost, and CatBoost algorithms outperformed others, with AUCs of 0.889, 0.883, and 0.864, respectively. Compared to previous studies that solely relied on morphological features, the application of radiomics yielded higher predictive accuracy, underscoring the significant contribution of radiomics features in assessing the risk of aneurysm rupture.

#### 3.2.2 Prediction of IA rupture using deep learning combined with multi-omics

Feng et al. (35) utilized a three-dimensional CNN to automatically detect and segment aneurysms, calculating 21 morphological features for each aneurysm. They extracted and identified 13 radiomics features related to aneurysm rupture, then used dimensionality reduction to construct SVM, RF, and MLP machine learning (ML) classification models to differentiate between ruptured and unruptured intracranial aneurysms (IAs). The results demonstrated that all three models exhibited high accuracy and were effective in discerning the status of aneurysms. Xie et al. (36) combined features extracted by CNN with radiomics features and patient clinical information, employing LASSO regression to select important feature variables for constructing an SVM-based aneurysm rupture risk prediction model. The accuracy and AUC were 89.78 and 89.09%, respectively. These studies confirmed that even with limited samples, the integration of CNN and radiomics can enhance model predictive performance. The optimal set of feature variables can provide essential biomarkers for determining rupture risk, which holds significant clinical implications for the personalized treatment planning of IAs. However, only age and gender were included as clinical features, necessitating further exploration of the impact of including additional clinical features on result accuracy. Turhon et al. (37) included 1,740 IA patients and constructed traditional ML and deep learning models based on clinical, radiomics, and morphological features. The results indicated that the deep learning-based radiomics model for predicting aneurysm rupture (AUC = 0.929) outperformed traditional ML models (AUC = 0.878), with the inclusion of morphological parameters also enhancing predictive performance. Unlike some studies that apply machine learning (ML) algorithms to morphological variables and hemodynamic parameters (38), Chen et al. (39) utilized computational fluid dynamics (CFD) to extract the hemodynamic cloud from 148 patients. They employed Point-Net to extract hemodynamic cloud features and combined these with morphological features to build integrated models using five ML and DL approaches. The results indicated that the use of the integrated model and the inclusion of hemodynamic cloud features can enhance the accuracy of IA rupture risk assessment. From the studies by Turhon and Chen, it’s evident that incorporating considerations of morphological and hemodynamic cloud features is crucial for improving model predictive performance, achieving more accurate classification results. This suggests that the multi-omics development in IAs is a feasible approach to further optimize and enhance model performance.

#### 3.2.3 Prediction of IA rupture using Transformer technology combined with deep learning

Li et al. (40) proposed an end-to-end deep learning method called TransIAR, designing a multi-scale 3D CNN to extract structural patterns of IAs and their surroundings. A Transformer encoder was used to learn spatial dependencies within the IA neighborhood, automatically learning morphological features from 3D-CTA data and accurately predicting the IA rupture status. The results demonstrated that the features learned by TransIAR were more effective and robust than manually crafted features, improving the accuracy of rupture status prediction by 10 to 15%, showcasing the superior performance of combining Transformer with CNN. Utilizing the attention mechanism of Transformers addresses the limitation of deep learning in long-distance modeling, demonstrating the superior performance of combining Transformers with CNNs. However, the study excluded aneurysms with a diameter smaller than 3 mm, which limits its clinical applicability.

### 3.3 Prediction of IA prognosis

With the development of embolization device technology, pipeline embolization devices (PEDs) have become an important tool for treating complex intracranial aneurysms (41). However, complications such as rebleeding and thromboembolic events can occur following IA treatment with PEDs, adversely affecting patient prognosis. Therefore, predicting potential outcomes before and after embolization is crucial for clinical decision-making (see Table 3).

**Table 3:** Brief summary of papers on the application of artificial intelligence and radiomics in the complication of intracranial aneurysms.

Author and year	Imaging modality	Dataset size and source	AI methodology	Model validation methods	Clinical outcomes predicted	Evaluation metrics
Bhurwani et al. (43)	DSA	163 patients, single center	DNN	Internal validation	Thrombosis	AUC = 0.77
Ma et al. (44)	DSA	64 patients, single center	Radiomics	Internal validation	Postinterventional rupture	Internal validation: AUC =0.912 External validation: AUC = 0.938
Jin et al. (47)	DSA	52 patients, single center	Radiomics	Internal validation	In-sent stenosis	AUC = 0.743

AUC, area under curve; DNN, deep neural networks.

#### 3.3.1 Prediction of postoperative complications based on radiomics

Angiographic parametric imaging (API), which relies on digital subtraction angiography (DSA), is a quantitative imaging tool that can extract hemodynamic-related parameters of contrast agent flow within IAs. This tool enables precise quantitative measurements and therapeutic evaluations of IAs. Radiomics can extract features from the ROI of API to predict prognosis. Liang et al. (42) screened 281 IAs treated with PEDs and analyzed the embolization results of 235 patients. Radiomic features were extracted from postoperative DSA images using API, and the Lasso algorithm was used to select radiomic features and calculate radiomics scores. The results suggested that API-derived radiomic features could be potential indicators for assessing the effectiveness of PED treatment for IAs. This novel approach provides new methods for predicting and evaluating the prognosis of PED treatment, such as combining API-derived radiomic features with deep learning to construct predictive models for patient prognosis, potentially offering more personalized clinical treatment options.

Bhurwani et al. (43) proposed the application of deep neural networks and DSA images for angiographic parametric imaging to predict the risk of thromboembolism after PED treatment. They analyzed DSA images before and after IA treatment to manually extract API parameters from the IA and corresponding aorta, standardizing them on projection views scanned before and after treatment. The results showed a prediction accuracy of 77.9% for thromboembolism after PED treatment, confirming the feasibility of using angiographic parametric imaging and deep neural networks to predict post-occlusion complications of IAs.

In addition to their individual applications, the combined use of radiomics and API (angiographic perfusion imaging) has shown great potential in predicting delayed intracranial aneurysm rupture post-surgery. Postoperative delayed rupture of intracranial aneurysms, a severe complication of pipeline embolization device (PED) treatment, poses a significant threat to patient outcomes. Therefore, accurately predicting the risk of rupture post-intervention is crucial. Liang et al. (42) extracted radiomic features from DSA images of patients after PED treatment using API and identified a higher radiomics score as a risk factor for complications. Ma et al. (44) utilized hemodynamic radiomic features derived from postoperative DSA angiography to quantitatively predict the risk of delayed rupture after PED treatment. For each patient, five perfusion parameter maps derived from post-intervention DSA were created, and radiomic features were obtained from each map. Six radiomic features were selected, and a radiomics score was calculated to predict the occurrence of IA rupture post-intervention. The results showed an AUC of 0.912 (95% CI: 0.767–1.000) for the training set and 0.938 (95% CI: 0.806–1.000) for the test set. The study confirmed the potential of using angiography-derived radiomic features to predict post-intervention intracranial aneurysm rupture, further expanding the application scope of radiomics. In summary, there is a significant association between radiomic features and occlusion and rupture of intracranial aneurysms in patients after PED treatment, but current research is still in its infancy, with relatively small sample sizes included in the studies. Additionally, given that API is inherently two-dimensional, its capacity to reflect the actual situation of three-dimensional aneurysms has limitations. However, the current workflow for quantifying API features requires manual delineation of the contrast medium’s entry into the target vessel and IA, a time-consuming and labor-intensive task that severely limits its application in clinical settings or large-scale studies. The application of deep learning in feature extraction can effectively address this issue. Podgorsak et al. (21) used CNNs to automate the extraction of radiomic features from angiographic parametric imaging analyses of IAs, comparing the results with manual extractions. The results demonstrated consistency between CNN and manual extractions, achieving minimal error in extracting API radiomic features and significantly reducing extraction time. This method enhances the clinical utility of using API features for prognosis prediction, potentially making it a tool to assist clinical decision-making.

Although post-pipeline embolization device (PED) treatment in-stent stenosis (ISS) is a self-limiting condition (45), its high incidence rate and subtle symptoms increase the risk of complications and can lead to severe consequences (46). The integration of morphological features and radiomics plays a significant role in predicting ISS after PED treatment. Jin et al. (47) extracted preoperative manually measured shape features and radiomic shape features, comparing patients who developed ISS with those who did not using follow-up DSA images. The results showed that one of the radiomic features, the elongation ratio, is an independent predictor of ISS. The study indicates that the more regular the shape of the aneurysm and the artery, the lower the likelihood of ISS.

Although clinical outcomes of aneurysms are influenced by various factors such as individual patient characteristics, severity of hemorrhage, different treatment modalities, and preoperative, intraoperative, and postoperative management (36), the application of radiomics and artificial intelligence in predicting the prognosis of patients with intracranial aneurysms offers new personalized treatment methods and holds great potential.

## 4 Discussion—existing challenges and future perspective

In recent years, artificial intelligence technology has been widely applied in the field of medical imaging, demonstrating tremendous potential in the automatic detection, status evaluation, and prognosis prediction of intracranial aneurysms (IAs). However, several challenges remain:

### 4.1 Algorithm interpretability

Local interpretable model-agnostic explanations (LIME) explores how slight perturbations to the input affect the output of a black-box model. By understanding these changes, LIME trains and builds a locally interpretable, agnostic model at the point of interest (the original input) to “explain” the black-box model (48). For example, when the model is used to predict the risk of an individual patient’s intracranial aneurysm rupture, LIME might discover that certain specific image regions (such as the location of the aneurysm) and the size of the intracranial aneurysm have a high positive weight in the model’s prediction. Clinicians can use the key features identified by LIME in the model’s predictions to understand why the model made such a prediction and combine this with their professional knowledge to make appropriate decisions.

SHapley Additive exPlanations (SHAP) provides more transparent result interpretations, calculating the importance of variables or features that make up the model and explaining how changes in important feature parameters affect the model’s predictive outcomes (49). By inputting features extracted from imaging, SHAP can provide a detailed feature contribution distribution through visualization, revealing which features are most important for predicting aneurysm rupture risk. SHAP helps doctors better understand how the model makes classifications or predictions based on image data, thus enhancing the reliability of the output results.

However, current interpretability algorithms still do not fully meet the needs of clinical doctors. Clinicians tend to use their medical knowledge to interpret the internal logic of how artificial intelligence algorithms use data to obtain predictive results, rather than relying on mathematically interpretable models. This might be because clinical data are inherently complex, and mathematically interpretable models overly focus on statistical characteristics of data, neglecting clinical realities, leading to inconsistencies between the interpretation results and clinical outcomes, and reducing clinicians’ trust.

To promote the clinical deployment of AI models, the primary interpretability approach for Transformers is based on attention weights. Additionally, an interactive radiology report diagnostic and evaluation system based on LLM can extend the detailed description of AI model detection results. For example, a study by Zhang et al. (50) developed an AI captioning system based on the Transformer’s BERT model, which autonomously provides prior fields for lesion descriptions. This greatly enhances the model’s interpretability by providing textual descriptions based on lesion details and is expected to directly output diagnostic results.

As the training costs of LLMs decrease, more LLM models for specific clinical purposes will be developed in the future. However, developers must be cautious about potential new and unpredictable behavior patterns or biases introduced by fine-tuning LLMs in open-source frameworks, which may exacerbate the “black box” phenomenon. To address these challenges, researchers need to adopt objective and unified evaluation metrics, explore new explanation methods, enhance the inherent interpretability of models, and promote innovative research in explainable algorithms.

### 4.2 Microaneurysm detection

Current research primarily focuses on the status detection and prognostic prediction of intracranial aneurysms (IAs). In previous studies on IA detection and diagnosis, there has been limited discussion and research specifically addressing microaneurysms (<3 mm), with the results of existing automatic segmentation and detection algorithms being relatively unsatisfactory. You et al. (19) reported that their VA-Unet model exhibited significant differences in segmentation performance for IAs of different sizes (*p* < 0.001), with poorer diagnostic performance for small aneurysms (<3 mm). In another study by Bo et al. (51), the GLIA-Net demonstrated a recall rate of 90.6% for aneurysms >7 mm but only a 70.3% recall rate for microaneurysms <3 mm. Additionally, a study (52) showed that the included CNN had an overall sensitivity greater than 98% (98–100%) for aneurysms >3 mm, whereas the overall sensitivity for aneurysms <3 mm was only 74.6%. Early screening of micro-aneurysms remains a clinical challenge. The emergence of the Vision Transformer (ViT) architecture and its application in medical image analysis offers a new method to address this issue. ViT-based models for disease image segmentation, such as TransUNet, Swin-UNet, and MedT, have demonstrated strong segmentation performance on images of other disease types (53, 54). ViT algorithms can divide an entire image into smaller image patches, using these patches’ linear embeddings as input for the Transformer network, and then employ supervised learning methods for image classification training. ViT utilizes the self-attention mechanism of Transformers to capture long-range dependencies between these patches, establishing richer contextual connections across the entire image. This integration of global information provides more precise analysis for identifying and locating micro-aneurysms with unclear boundaries compared to traditional CNNs, which rely on local receptive fields.

### 4.3 Training data and clinical applicability

AI and radiomics dependence on extensive imaging data. Current research is predominantly based on small samples and single-center studies. Faced with limited datasets, most studies use CNN networks for feature extraction and traditional ML for classification and prediction, combining the early stages of deep learning with the latter stages of radiomics. Models may suffer from overfitting or underfitting due to overtraining. Most models lack external validation, making it difficult to judge their generalizability across patient populations with different statistical characteristics, and their experimental results require further verification. Currently, research on large language models and Transformer architectures in the medical field is gradually underway, requiring significantly larger datasets for training than traditional CNNs or ML to achieve good model performance. High-quality, large-sample datasets enable models to mine and learn deeper data features and patterns, enhancing model accuracy. Additionally, the data should be more diverse, including various types of intracranial aneurysms, such as microaneurysms and cases of arterial stenosis, while ensuring a balance between positive and negative samples. Challenging cases should not be excluded merely to achieve numerical performance breakthroughs, to better reflect clinical reality and enhance model generalizability. The neuroimaging field currently lacks large, publicly accessible datasets like the cancer imaging TCIA and genomic GEO databases for researchers. Thus, establishing large, multicenter databases and international collaboration to collect and share patient demographics, aneurysm radiographic characteristics, imaging methods, and imaging parameters is essential for advancing AI.

The clinical applicability of predictive models in AI and radiomics remains limited. Studies have shown that AI models often exhibit insufficient generalizability in external validations, manifesting as performance declines (55), possibly related to patient ethnic differences, medical conditions, and other factors. Even AI decision support systems approved for clinical use face questions about their safety and efficacy in different clinical environments. Therefore, conducting prospective, multicenter, randomized controlled clinical trials to assess the reliability of AI and radiomics models in radiographic image analysis and ensure their benefit for patient prognosis is necessary. Continual model algorithm updates and comprehensive clinical research are also crucial, oriented towards the needs of patients and healthcare workers, to optimize models and simplify processes, reducing the usage threshold for healthcare workers. Developing multi-task AI models to achieve a fully automated service for the detection, segmentation, status assessment, and prognostic prediction of intracranial aneurysms, and integrating radiomics with patients’ other clinical histories, laboratory indicators, and past report results using LLMs can aid physicians in designing accurate and personalized treatment plans. Standardizing operation processes and providing data governance, model deployment, and maintenance services are also essential.

### 4.4 Ethical considerations

Privacy protection is one of the main ethical challenges in the application of AI and radiomics in the medical field. Patient data, containing highly sensitive personal information, must be rigorously protected. Federated learning, an innovative machine learning architecture, provides a new method for multi-party data collaboration while safeguarding privacy. In this framework, a virtual model is designed to enable multi-center data collaboration without exchanging data (56). Each center trains the model on local devices and then transmits the model updates to a central server, not the original data. The central server aggregates these model updates from different devices to enhance the performance of the global model and returns it to each device for further local training and optimization. Under the federated mechanism, since the original data is not transferred, the risks of data leakage and misuse are significantly reduced by avoiding data transmission and centralized storage. This approach effectively allows multiple institutions to use data and build models while adhering to user privacy protection, data security, and government regulations.

Algorithmic bias is also a significant ethical issue in the medical AI field, often facing problems such as data bias, label bias, algorithm design bias, and data collection method bias, which can lead to AI models providing incorrect suggestions, influencing doctors’ judgments. Medical decisions are crucial for patients, and incorrect judgments can endanger their lives. However, studies have shown that some healthcare professionals heavily rely on suggestions provided by AI-assisted decision systems, sometimes unable to recognize incorrect recommendations, leading to severe consequences. Medical professionals should always maintain critical thinking towards AI system suggestions and bear ultimate responsibility in final decisions. Relevant laws and regulations should also further refine the determination and division of responsibility.

## 5 Conclusion

This study provides a comprehensive review of the application of artificial intelligence and radiomics in the detection, segmentation, and status recognition prediction of intracranial aneurysms, as well as postoperative prognostic prediction, showcasing the potential of AI and radiomics in this field. The development of new technologies, such as large language models (LLMs) and self-supervised learning, is expected to further advance research in this domain. Future studies should focus more on the practical application of the constructed models, transitioning AI and radiomics from research to clinical practice. This transition necessitates a collaborative effort by global researchers to establish standard databases, enhance model performance, and pay particular attention to model interpretability. With advancements in big data technology and the proliferation of precision medicine concepts, the integration of medical imaging, artificial intelligence, and radiomics is poised to contribute significantly to the field. They are expected to jointly create a clinical decision support system, offering more personalized and precise treatment options for patients with intracranial aneurysms, improving treatment outcomes and quality of life, and collectively advancing the field of precision medicine.

## Author contributions

ZW: Writing – original draft, Writing – review & editing. YW: Writing – original draft, Writing – review & editing. YZ: Writing – original draft, Writing – review & editing. YH: Writing – original draft. CY: Writing – original draft. YP: Supervision, Writing – review & editing. XZ: Supervision, Writing – review & editing. PZ: Supervision, Writing – review & editing. ZZ: Funding acquisition, Supervision, Writing – review & editing.
